# Inhibition of Histone H3K9 Acetylation by Anacardic Acid Can Correct the Over-Expression of Gata4 in the Hearts of Fetal Mice Exposed to Alcohol during Pregnancy

**DOI:** 10.1371/journal.pone.0104135

**Published:** 2014-08-07

**Authors:** Chang Peng, Jing Zhu, Hui-Chao Sun, Xu-Pei Huang, Wei-An Zhao, Min Zheng, Ling-Juan Liu, Jie Tian

**Affiliations:** 1 Heart Centre, Children's Hospital of Chongqing Medical University, Chongqing, China; 2 Key Laboratory of Pediatrics in Chongqing, Chongqing, China; 3 Ministry of Education Key Laboratory of Child Development and Disorders, Chongqing, China; 4 Chongqing International Science and Technology Cooperation Center for Child Development and Disorders, Chongqing, China; 5 Department of Biomedical Science, Charlie E. Schmidt College of Medicine, Florida Atlantic University, Boca Raton, Florida, United States of America; Northwestern University, United States of America

## Abstract

**Background:**

Cardiovascular malformations can be caused by abnormalities in *Gata4* expression during fetal development. In a previous study, we demonstrated that ethanol exposure could lead to histone hyperacetylation and *Gata4* over-expression in fetal mouse hearts. However, the potential mechanisms of histone hyperacetylation and *Gata4* over-expression induced by ethanol remain unclear.

**Methods and Results:**

Pregnant mice were gavaged with ethanol or saline. Fetal mouse hearts were collected for analysis. The results of ethanol fed groups showed that global HAT activity was unusually high in the hearts of fetal mice while global HDAC activity remained unchanged. Binding of P300, CBP, PCAF, SRC1, but not GCN5, were increased on the *Gata4* promoter relative to the saline treated group. Increased acetylation of H3K9 and increased mRNA expression of *Gata4*, *α-MHC*, *cTnT* were observed in these hearts. Treatment with the pan-histone acetylase inhibitor, anacardic acid, reduced the binding of P300, PCAF to the *Gata4* promoter and reversed H3K9 hyperacetylation in the presence of ethanol. Interestingly, anacardic acid attenuated over-expression of *Gata4*, *α-MHC* and *cTnT* in fetal mouse hearts exposed to ethanol.

**Conclusions:**

Our results suggest that P300 and PCAF may be critical regulatory factors that mediate *Gata4* over-expression induced by ethanol exposure. Alternatively, P300, PCAF and Gata4 may coordinate over-expression of cardiac downstream genes in mouse hearts exposed to ethanol. Anacardic acid may thus protect against ethanol-induced *Gata4*, *α-MHC*, *cTnT* over-expression by inhibiting the binding of P300 and PCAF to the promoter region of these genes.

## Introduction

In children, congenital heart disease (CHD) accounts for nearly one-third of all major congenital anomalies [1], a major cause of death in infants of 1 year of age or below [2]. Cardiac development is a very complex process, regulated by both genetic and epigenetic pathways. Alcohol consumption is a common teratogenetic factor that is thought to affect epigenetic regulation of embryonic development and contribute to CHD [3]–[6]. Alcohol consumption during pregnancy is associated with multiple cardiovascular malformations [7]. However, the potential mechanism of CHD induced by alcohol is poorly understood.

Many studies indicate that the *Gata4* transcription factor plays a critical role during cardiogenesis, from primitive heart tube to maturation i.e. the four-chambered heart [8]–[11]. Both genetics and epigenetics play a part in regulating the expression of *Gata4* and alterations that lead to uncontrolled expression of *Gata4* could affect normal development of the heart. We have demonstrated that high consumption of alcohol or its metabolites can induce histone hyperacetylation that leads to over-expression of *Gata4* [12], [13]. However, it is not clear whether alcohol exposure increases myocardial histone acetylation or *Gata4* over-expression in developing hearts. Furthermore, it is not understood which isoforms of HATs and HDACs take part in *Gata4* regulation during cardiogenesis.

Here, we examined the impact alcohol exposure has on HAT and HDAC activity during heart development, and explored the effects of P300, CBP, PCAF, SRC1 and GCN5 on *Gata4* over-expression. We present data on the effects of alcohol on cardiac downstream genes *α-MHC*, *cTnT*, *α-actin*. Finally, we tested if a pan-histone acetylase inhibitor, anacardic acid, has a protective effect on alcohol-induced *Gata4* and cardiac downstream gene over-expression.

## Materials and Methods

### Treatment of Mice

Pathogen free male and female, 9 to 11 week old Kunming mice (20–25 g) were purchased from the Experiment Animal Center of Chongqing Medical University (Chongqing, China). All the trials on the animals selected for the experiments were approved by the Animal Care and Use Committee at the Chongqing Medical University. Mice were housed and allowed food *ad libitum*, and maintained in a controlled environment (22±1°C, 55±5% humidity) with a 12 h: 12 h light: dark cycle. Mated female mice were examined for a vaginal plug in the morning. If a vaginal plug was observed, embryos were considered to be (E) 0.5 day. Pregnant mice were randomly assigned to be gavaged with 56% ethanol (control groups received equivalent normal saline), In some cases, ethanol treated pregnant mice were administered the pan-histone acetylase inhibitor anacardic acid. Anacardic acid was dissolved in sterile DMSO at a concentration of 1 mg/ml and stored at 4°C. Ethanol (56% v/v) was fed daily to pregnant dams at a volume of 5 ml per kilogram per day from E8.5–E16.5 by gavage. Anacardic acid was administered by intraperitoneal injection at a dose of 5 mg per kilogram per day during days E8.5–E16.5. On days, E14.5 and E16.5, pregnant mice were euthanized using carbon dioxide narcosis and embryonic hearts were isolated from pups. Hearts from 0.5 day old and 7 day old neonatal mice were collected, as well.

### HAT and HDAC Activity Assays

After homogenization of cardiac tissues, nucleoproteins were extracted using a Nuclear Extract Kit (Merck Millipore, DA, GER) according to the manufacturer's instructions. HAT and HDAC activities of the nuclear protein extracts were determined using a colorimetric assay included in the HAT and HDAC assay kits (GenMed Scientific Inc, Shanghai, China).

### Chromatin Immunoprecipitation (ChIP) Assay

After homogenization of cardiac tissues, formaldehyde (1%) was added to the samples to cross-link protein-DNA complexes. ChIP trials were conducted by use of a ChIP assay kit (Merck Millipore, DA, GER). After cross-linking, the DNA was fragmented by sonication, this consisted of 20 cycles of 30 seconds each time with an interval of 30 seconds to cool down. Then protein-DNA complexes were precipitated by monoclonal anti-P300 antibody, anti-CBP antibody, anti-PCAF antibody, anti-SRC1 antibody (ChIP grade, Abcam, Cambridge, UK), anti-GCN5 antibody (ChIP grade, Epigentek, NY, USA) or polyclonal anti-H3AcK9 antibody (ChIP grade, Abcam, Cambridge, UK) respectively, DNA was extracted using a DNA purification kit (Merck Millipore, DA, GER). The experiment contained both a positive control group (precipitated by anti-RNA polymerase II antibody) and a negative control group (precipitated by normal mouse IgG). Specific primers were designed for recognizing the promoter of *Gata4*, *cTnT*, *α-MHC* and *α-actin* in quantitative Real-Time PCR assays, to ascertain cardiac development-related genes, which may interact with HAT proteins and be regulated by HATs. The sequences of specific primers were as follows: *Gata4* sense primer: 5′-TCTTCCACTTCCACACGTACCAA-3′ and antisense primer: 5′-CAGAGGGAGTTGGGAGACGTAG-3′; *cTnT* sense primer: 5′ -TAACAGTGTCTGGAAGCGTCA-3′ and antisense primer: 5′-CAGAGTGACTGGCACAAGGT-3′; *α-MHC* sense primer: 5′-AGGACAGGGGTTGCCTCT-3′ and antisense primer: 5′-AGGTGCTGCTTTGAATGCC-3′; *α-actin* sense primer: 5′-TGCCTCAGCCCCCTCTAG-3′ and antisense primer: 5′-GCAGACAACTGGTGGAAGAG-3′; *RPL13A* sense primer: 5′-GAAAGCCTTGTCGCATCCCT-3′ and antisense primer: 5′-GAAAGCCAAAGCTGGATGACA-3′. Annealing temperatures were as follows: 59°C for *Gata4*, 57°C for *cTnT*, *α-MHC*, *α-actin* and *RPL13A*. PCR products were detected by 2% agarose gel electrophoresis. The quantity of DNA immunoprecipitation by each HAT antibody reflected the HAT isoforms that were bound to the promoters.

### Western Blot Analysis

Mouse hearts were collected, and nucleoproteins were extracted as above. Nucleoproteins were separated and electrophoresed on 12% sodium dodecyl sulfate (SDS) polyacrylamide gels and then transferred to polyvinylidene difluoride (PVDF) membrane (Merck Millipore, DA, GER). After incubation with 5% non-fat milk for 1 h, the blots were probed with rabbit polyclonal antibodies against acetylated groups of histone H3K9 (Abcam, Cambridge, UK, 1∶1,000 dilution) or rabbit polyclonal antibody against histone H3 (Beyotime, Shanghai, China, 1∶1,000 dilution) in Tris Buffered Saline with Tween 20 (TBST) plus 5% non-fat milk at 4°C overnight. HRP conjugated goat anti-rabbit antibody (Santa Cruz Biotechnology, CA, USA) was used as the secondary antibody. After scanning, bands were subjected to analysis using Quantity One Version4.4 software (Bio-Rad, CA, USA). Western blot experiments were repeated six times to confirm the results.

### Total RNA Extraction and Quantitative RT-PCR

Total RNA was extracted from the collected cardiac tissues using an RNA extraction kit (Bioteke, Beijing, China). First-strand cDNA was synthesized from 500 ng to 1,000 ng RNA by using oligo dT-Adaptor primers and AMV reverse transcriptase (Takara, Dalian, Liaoning, China) following the manufacturer's instruction. Then cDNA was amplified with gene-specific primers (Shanghai DNA biotechnologies, Shanghai, China) and SYBR Green dye kit (Takara, Dalian, Liaoning, China). The primer sequences of cardiac-specific gene and control were designed as follows: *Gata4* sense primer: 5′-TGCCAACTGCCAGACTACCAC-3′ and antisense primer: 5′-TCAGGTTCTTGGGCTTCCGT-3′; *α-MHC* sense primer: 5′ -TGAGACGGATGCCATACAGA-3′ and antisense primer: 5′-GCAGCCTGTGCTTGGTCTT-3′; *cTnT* sense primer: 5′-GAAGGAAAGGCAGAACCGC-3′ and antisense primer: 5′-GCCTCCAGGTTGTGAATACTC-3′; *α-actin* sense primer: 5′-TGCTGTCCCTCTATGCTTCC-3′ and antisense primer: 5′-GCTGTGGTCACGAAGGAATAG-3′; *β-actin* sense primer: 5′-CCTTTATCGGTATGGAGTCTGCG-3′ and antisense primer: 5′-CCTGACATGACGTTGTTGGCA-3′. Annealing temperatures were as follows: 59°C for *Gata4* and *β-actin*, 56°C for *α-MHC*, *cTnT* and *α-actin*. The analysis of relative mRNA expression was carried out using 2^−ΔΔCt^ method [14], and *β-actin* was used as an endogenous housekeeping gene to normalize the mRNA level.

### Statistical Analysis

The data was presented as means ± standard deviation while the statistical evaluations were performed using independent-samples by applying T-test, continuity correction chi-square test and one-way ANOVA. A p-value <0.05 was considered to be statistically significant for all analyses.

## Results

### Effects of alcohol exposure on HAT and HDAC activity

**Figure 1:**
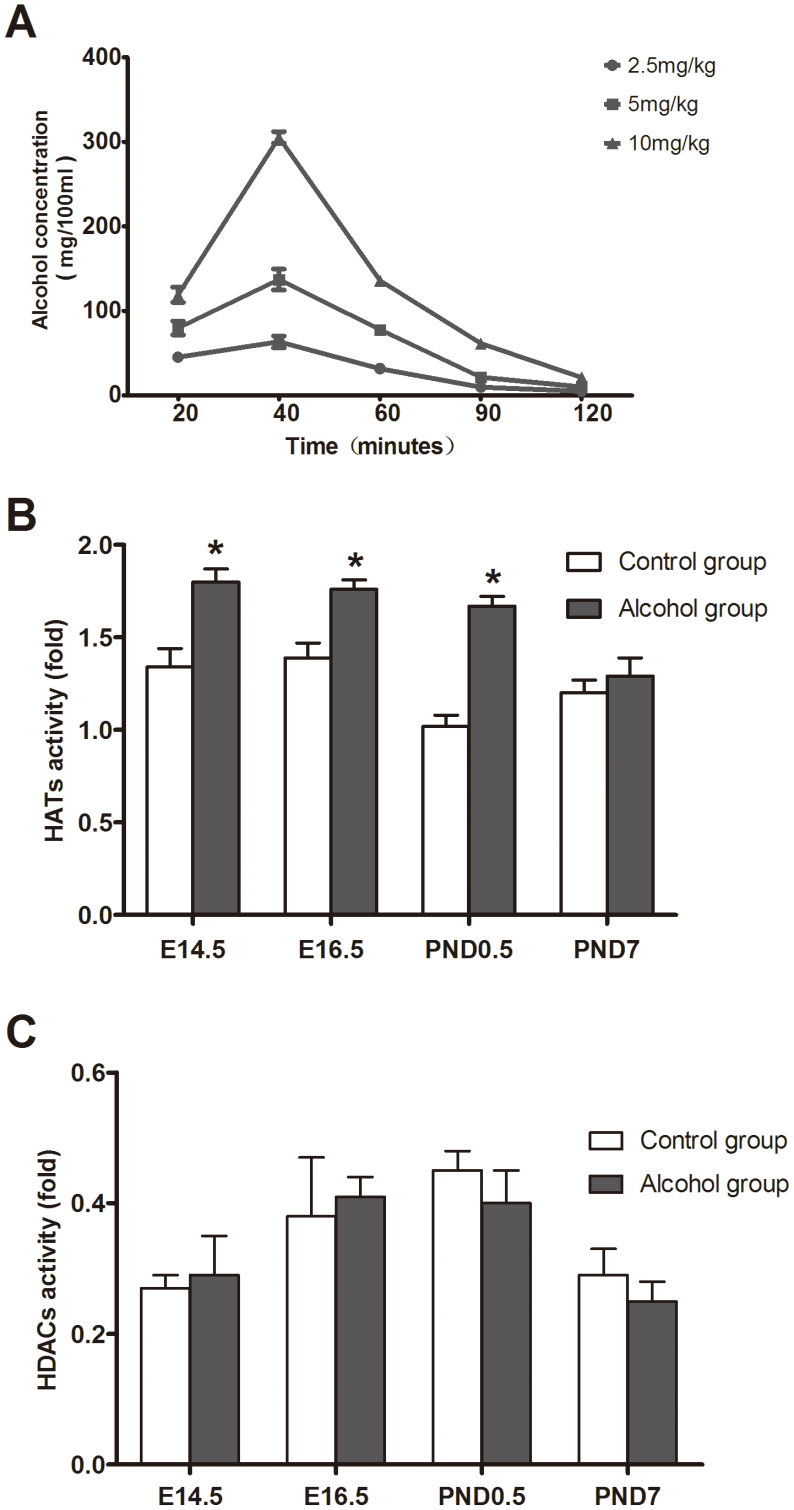
Effects of alcohol exposure on activities of HAT and HDAC. To analyze the impact of alcohol on activities of HAT and HDAC in cardiac tissues, different doses of alcohol were used to choose optimal exposure dose in pregnant mice. The blood-alcohol concentration (A) after gavaging with different doses of 56% ethanol in mice (n = 6). The alcohol stress (56%) increases HAT activity (B) in E14.5, E16.5 and PND0.5, while it remained unchanged in PND7 and no any effects observed on HDAC activity (C) in myocardial tissues. *: P<0.05 vs. control group (n = 9). E14.5: embryo 14.5 day, E16.5: embryo 16.5 day, PND0.5: postnatal day 0.5, PND7: postnatal day 7.

To determine the effect of alcohol on histone acetyltransferases (HAT) and histone deacetylases (HDAC), we first ascertained an optimal alcohol exposure dose. Pregnant mice were intragastrically administrated with different volumes of 56% (v/v) ethanol in water to find the optimal dose. For this purpose, 5 ml/kg for alcohol exposure dose was selected according to the blood-alcohol concentration (**Figure 1 A**). Alcohol related side effects were observed (**Table 1**). The maximum blood-alcohol concentration was 137.1 mg/100 ml after 40 minutes of the intragastric administration of 5 ml/kg alcohol.

**Table 1:** Side effects caused by different dose of alcohol.

Alcohol dose (mg/kg)	Total	Abortions	Stillbirths	Intestinal tympanites	Normal
2.5	20	0 (0%)	0 (0%)	1 (5%)	19 (95%)
5	20	1 (5%)	1 (5%)	1 (5%)	15 (85%)
10	20	6 (30%)	3 (15%)	2 (10%)	9 (45%)

The results of HAT assay showed that alcohol could increase significantly the total HAT activity in heart tissue from the fetal mice exposed to alcohol on E14.5, E16.5 and postnatal day 0.5 (PND0.5) compared to control groups (P<0.05) (**Figure 1 B**). However, HDAC activity was not altered in heart tissue from the fetal mice exposed to alcohol compared to the controls on E14.5, E16.5, PND0.5 and PND7 (**Figure 1 C**). This data suggested that the alcohol enhances selectively HAT activity rather than affecting HDAC activity in cardiac tissues from fetal mice exposed to alcohol.

### HATs were involved in acetylation on different sites of the *Gata4* promoter

**Figure 2:**
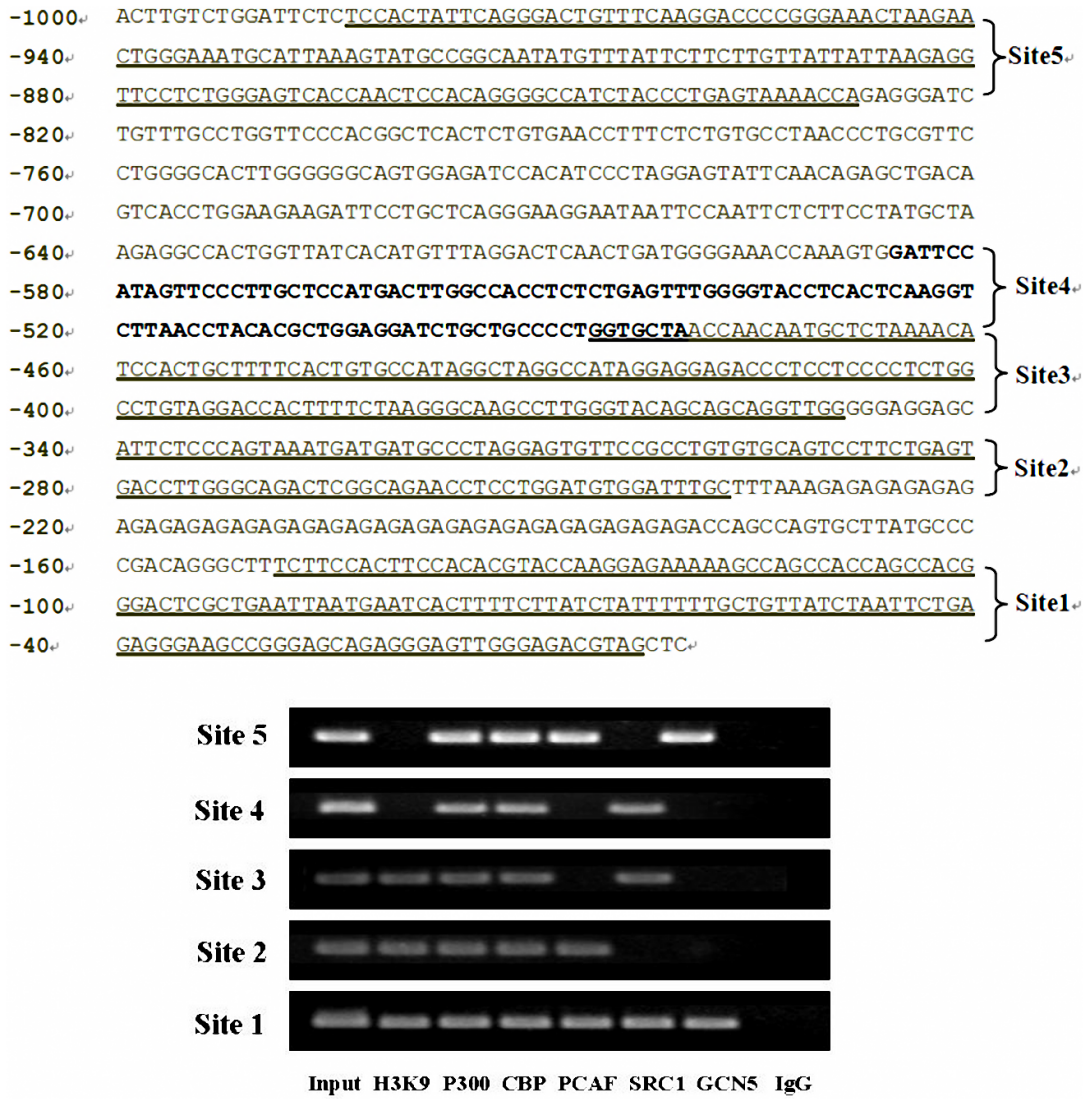
Acetylation sites of HATs on the *Gata4* promoter. The sequence of 1.0 kb at up-stream of the transcription start site of mouse *Gata4* is indicated in Upper and band density is in the bottom. ChIP-PCR results run on agarose gel electrophoresis showed that five HATs (P300, CBP, PCAF, SRC1 and GCN5) proteins could bind to multiple sites on the promoter of *Gata4* for possible acetylation of histone H3K9 on this site. All the five HATs (P300, CBP, PCAF, SRC1 and GCN5) were amplified with DNA fragments precipitated by anti-P300, anti-CBP, anti-PCAF, anti-SRC1 and anti-GCN5 antibodies. H3K9 and IgG were amplified with DNA fragment precipitated by anti-H3AcK9 antibody during the later with normal mouse IgG. Site 1: 43801992 bp∼43802137 bp, Site 2: 43802226 bp∼43802328 bp, Site 3: 43802338 bp∼43802475 bp, Site 4: 43802469 bp∼43802574 bp, Site 5: 43802817 bp∼43802972 bp.

We investigated the regulatory relationship between HATs (P300, CBP, PCAF, SRC1 and GCN5) and histone acetylation status on the promoter of cardiac nuclear transcription factor *Gata4*. To do this, we isolated cardiac tissues from normal fetal mice on E14.5 to detect the binding of HATs on the promoter region of *Gata4* using PCR assays following ChIP. Sequence analysis revealed several putative regulatory domains (H3K9 acetylation sites) and multiple HAT binding sites (P300 binding sites, CBP binding sites, PCAF, SRC1, GCN5, etc.). P300 and CBP could bind to sites 1, 2, 3, 4 and 5; PCAF could bind to sites 1, 2 and 5; SRC1 could bind to sites 1, 3 and 4; while GCN5 could bind to sites 1 and 5 only. It is noteworthy that the binding site of HATs (P300, CBP, PCAF, SRC1 and GCN5) and the site of histone H3K9 acetylation within the first 150 bp of the transcription start site were critical for the gene expression (**Figure 2**).

### Alcohol exposure induces hyperacetylation of histone H3K9 in the developing heart

**Figure 3:**
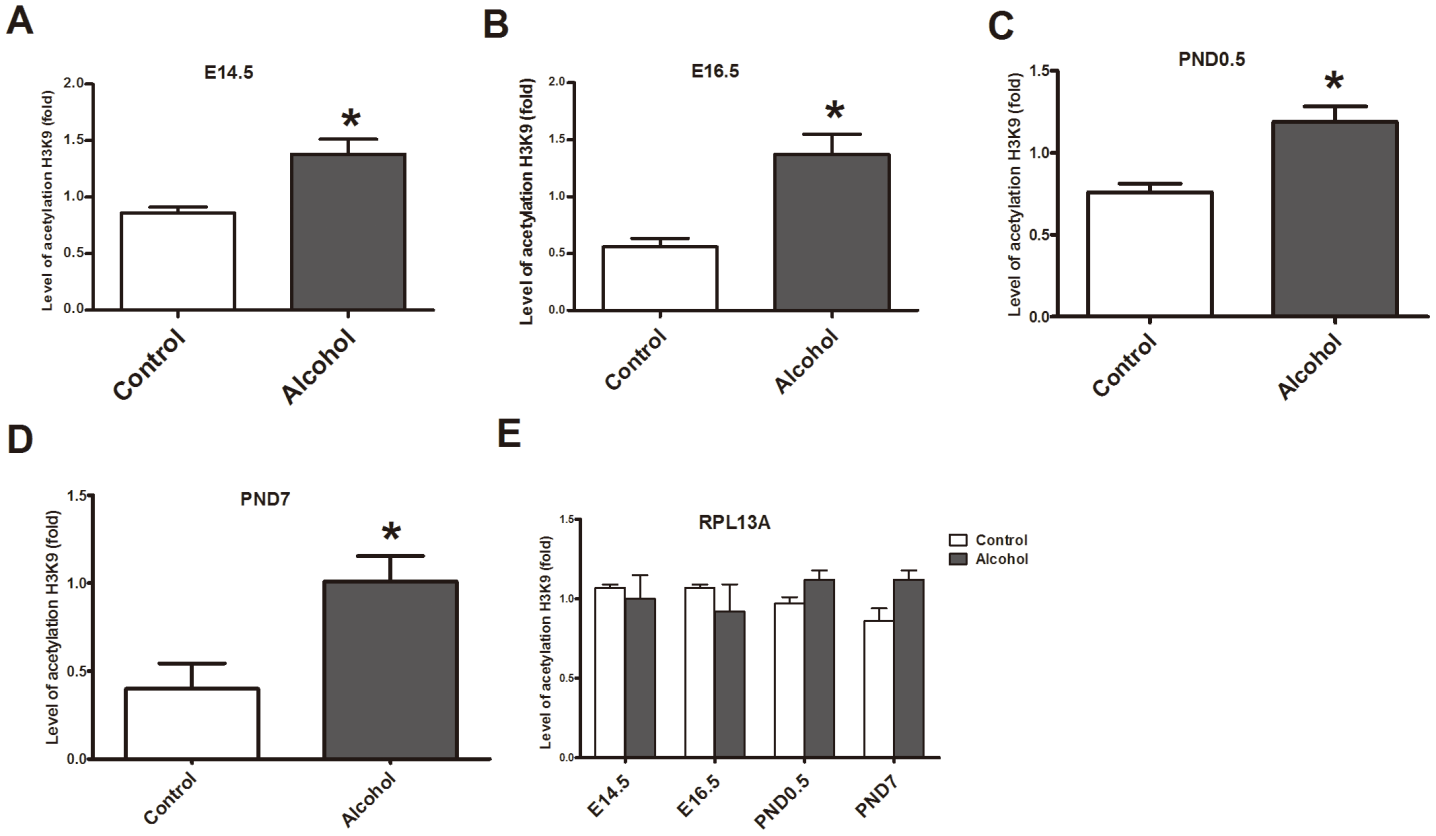
Hyperacetylation of histone H3K9 induced by alcohol on the *Gata4* promoter. ChIP-Q-PCR results (A,B,C,D) showed that alcohol is linked to histone H3K9 hyperacetylation on the *Gata4* promoter at E14.5, E16.5, PND0.5 and PND7, respectively. However, alcohol could not affect acetylation of H3K9 on the promoter of *RPL13A* (E). *: P<0.05 vs. control group (n = 3). E14.5: embryo 14.5 day, E16.5: embryo 16.5 day, PND0.5: postnatal day 0.5, PND7: postnatal day 7.

**Figure 4:**
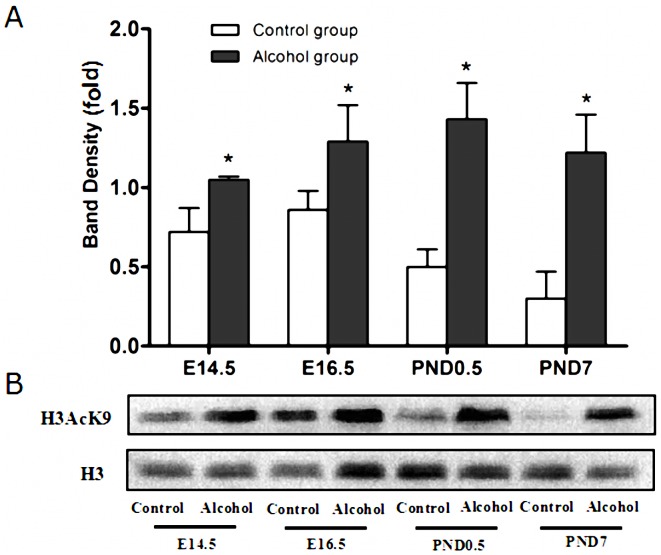
Histone H3K9 hyperacetylation induced by alcohol. According to the western blot analyses, the level of acetylation of histone H3K9 increased significantly after alcohol exposure with 5/kg on E14.5, E16.5, PND0.5 and PND7. *: P<0.05 vs. control group (n = 6). (A): statistic analysis, (B): band density. E14.5: embryo 14.5 day, E16.5: embryo 16.5 day, PND0.5: postnatal day 0.5, PND7: postnatal day 7.

The level of H3K9 acetylation was analyzed using Q-PCR after ChIP. The results of ChIP-Q-PCR showed that fetal mice exposed to alcohol on E14.5, E16.5, PND0.5 and PND7 (P<0.05 vs. control group) exhibited hyperacetylation of histone H3K9 on the promoter of *Gata4* in cardiac tissue (**Figure 3**). Western blot data showed that the acetylation of histone H3K9 in the alcohol treated group was increased significantly compared to that in control groups at these stages (**Figure 4**).

### Effects of alcohol on binding of HATs to the promoter of *Gata4* in cardiac tissue

**Figure 5:**
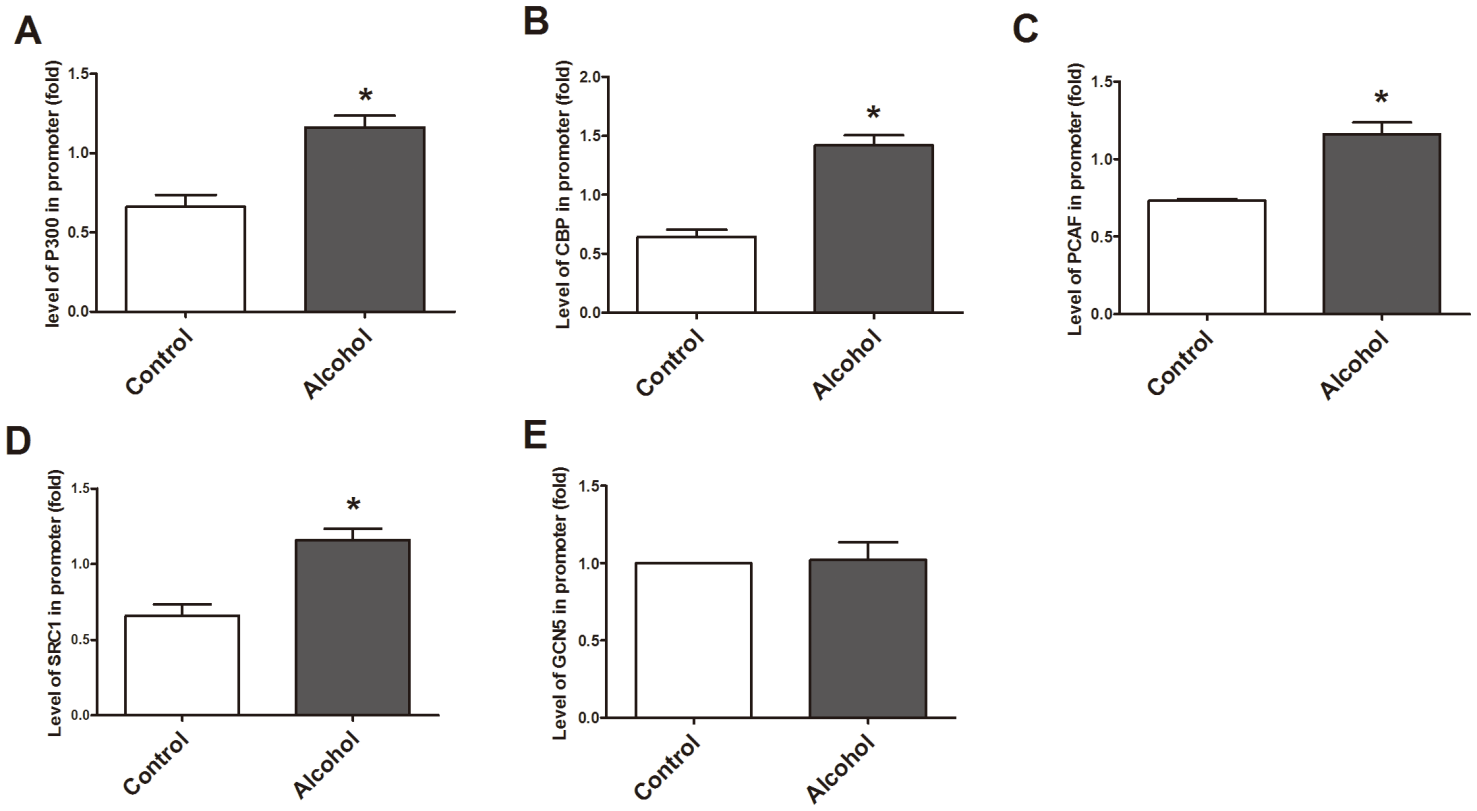
Effect of alcohol on binding of P300, CBP, PCAF, SRC1, GCN5 to the *Gata4* promoter. According to ChIP-Q-PCR results, alcohol could increase binding of P300, CBP, PCAF, SRC1 to the *Gata4* promoter on E14.5, whereas binding of GCN5 remained unchanged to the promoter region. *: P<0.01 vs. control group (n = 3). A–E was amplified with DNA-fragment precipitated with their respective antibodies i.e. anti-P300, anti-CBP, anti-PCAF, anti-SRC1 and anti-GCN5 antibody.

ChIP-Q-PCR data indicated that alcohol exposure could significantly increase the binding of P300, CBP, PCAF and SRC1 to the *Gata4* promoter at E14.5 (P<0.01) vs. control group (**Figure 5 A–D**). However, the binding of GCN5 on the *Gata4* promoter in the same cardiac samples had no significant change compared to the control group at E14.5 (**Figure 5 E**), suggested that P300, CBP, PCAF and SRC1 may have a regulatory function for the acetylation of this regulatory site (the first 150 bp upstream of the transcription start site) that can effect *Gata4* gene expression.

### Anacardic acid reduces hyperacetylation of H3K9 induced by alcohol exposure

**Figure 6:**
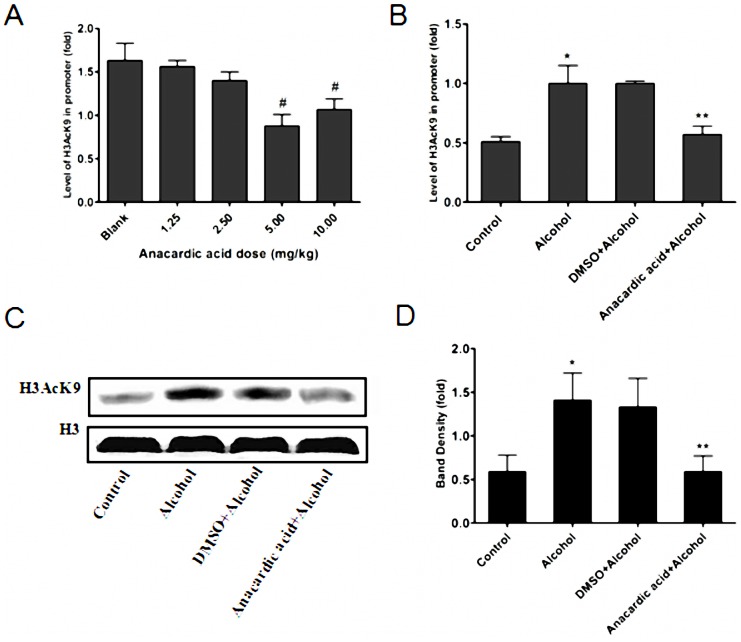
Anacardic acid inhibits histone H3K9 hyperacetylation under alcohol treatment. Pregnant mice were exposed to various portions (0, 1.25, 2.5, 5 and 10 mg/kg) of anacardic acid to ascertain optimal dose. The result showed that the level of H3AcK9 on the *Gata4* promoter after intraperitoneal injection with 5 mg/kg anacardic acid was the lowest compared with another dose. ^#^: P<0.05 vs. blank group (n = 3) (A). ChIP-Q-PCR results further confirms that anacardic acid (5 mg/kg) could reduce significantly hyperacetylation of histone H3K9 induced by alcohol on the *Gata4* promoter at E14.5. *: P<0.01 vs. control group (n = 3), **: P<0.01 vs. alcohol group (n = 3) (B). Western blot analysis showed that the anacardic acid (5 mg/kg) could inhibit significantly hyperacetylation of histone H3K9 in the myocardium. *: P<0.01 vs. control group (n = 6), **: P<0.01 vs. alcohol group (n = 6). (C): band density, (D): statistic analysis.

To evaluate the inhibition of anacardic acid *in vivo*, the optimal exposure dose should first be established. Pregnant mice were intraperitoneally injected with anacardic acid at different dosages (0, 1.25, 2.5, 5, 10 mg/kg) based on reports in the literature [15]. The optimal dose (5 mg/kg) was ascertained according to the level of H3AcK9 on the promoter of *Gata4* (**Figure 6 A**). Pregnant mice were treated by anacardic acid at this dose to confirm the inhibitory effect of anacardic acid on H3K9 hyperacetylation induced by alcohol. ChIP-Q-PCR results showed that the pan-histone acetylase inhibitor anacardic acid reduced significantly the hyperacetylation of H3K9 on the promoter region of *Gata4* from the fetal mouse hearts in alcohol given groups (**Figure 6 B**). Western blot data showed that anacardic acid could significantly decrease hyperacetylation of H3K9 induced by alcohol (P<0.01) vs. the alcohol alone group (**Figure 6 C, D**). In addition, we also observed that the ratios of abortions, stillbirths and intestinal tympanites declined slightly in mice treated with anacardic acid (**Table 2**).

**Table 2:** Effects of anacardic acid on alcohol related side effects in pregnant mice exposed to alcohol.

Side effects	Alcohol group (n = 30)	Alcohol+anacardic acid group (n = 35)	χ^2^ value.	*P* value
Abortions	3 (10.00%)	1 (2.86%)	0.458	0.498
Stillbirths	2 (6.67%)	1 (2.86%)	0.019	0.891
Intestinal tympanites	2 (6.67%)	1 (2.86%)	0.019	0.891

### Anacardic acid inhibits binding of P300, PCAF to the promoter of *Gata4*

**Figure 7:**
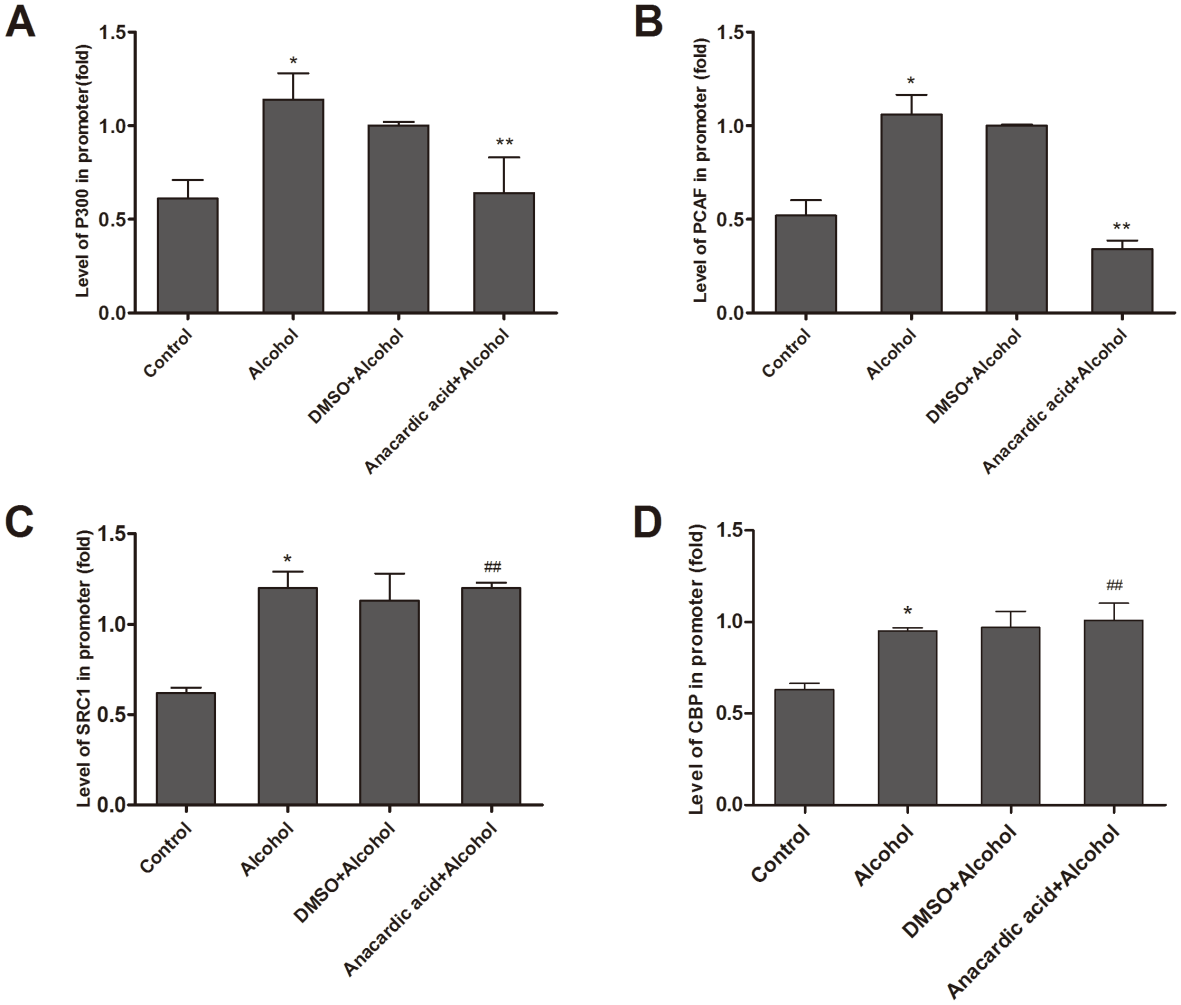
Anacardic acid inhibits the binding of HATs to the *Gata4* promoter. Alcohol could improve binding of P300, PCAF, SRC1 and CBP to the *Gata4* promoter on E14.5, and anacardic acid could repress binding of P300 and PCAF to the *Gata4* promoter in cardiac tissues whereas anacardic acid could not decrease binding of SRC1 and CBP to the *Gata4* promoter in the same cardiac tissues (A,B,C,D). *: P<0.01 vs. control group (n = 3), ^##^: P<0.05 vs. alcohol group (n = 3). **: P<0.01 vs. alcohol group (n = 3).

Our results indicated that the anacardic acid could reduce significantly the binding of P300 and PCAF to the promoter region of *Gata4* in fetal hearts exposed to alcohol (**Figure 7 A, B**). However, binding of SRC1 and CBP to the promoter region of *Gata4* had no change in the same tested cardiac tissues (**Figure 7 C, D**). These data suggest that P300 and PCAF may play a key role in regulating histone H3K9 hyperacetylation of *Gata4*.

### Anacardic acid down-regulates mRNA over-expression of *Gata4* induced by alcohol

**Figure 8:**
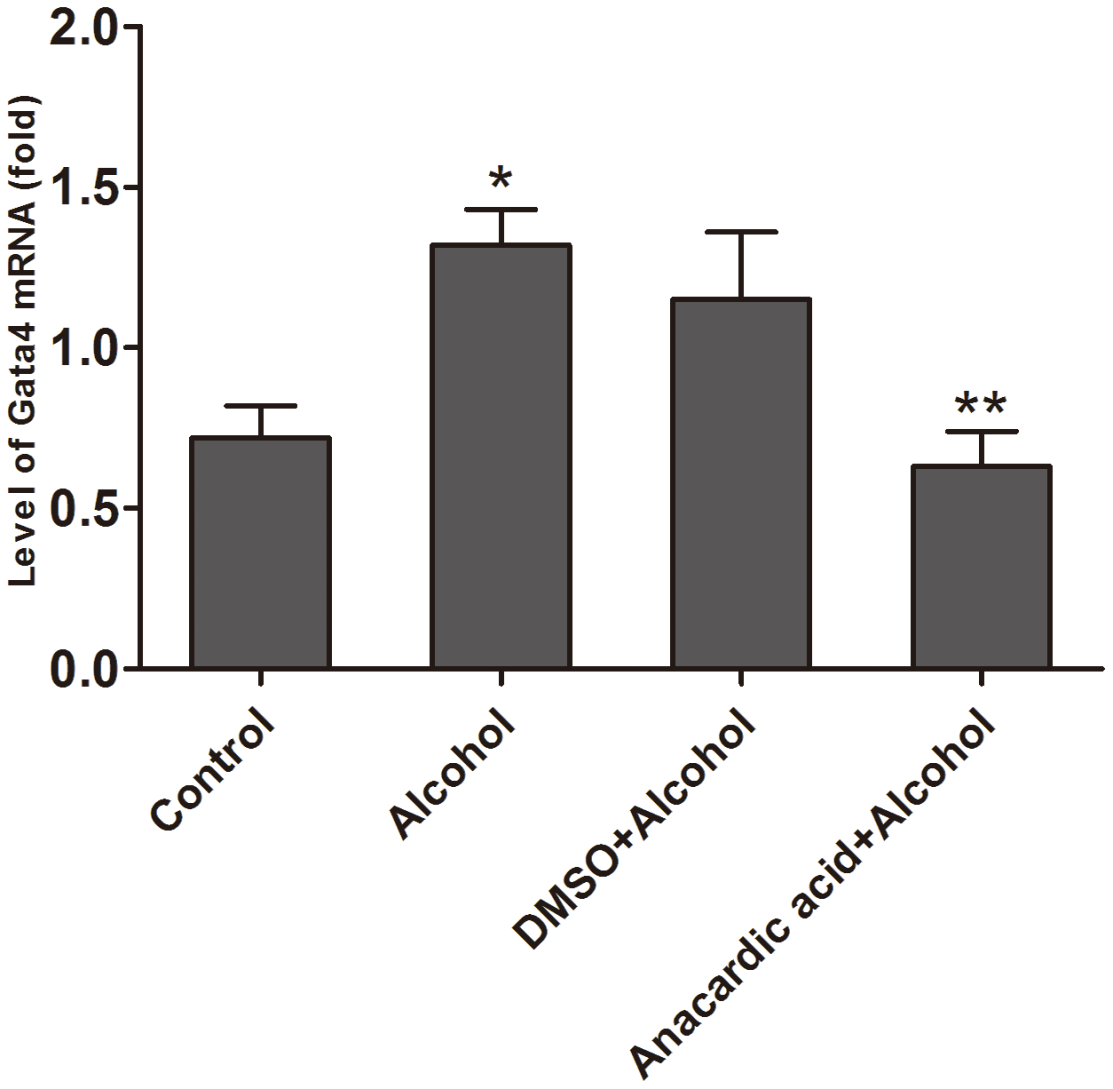
Anacardic acid inhibits the over-expression of *Gata4* mRNA induced by alcohol. The mRNA expression of *Gata4* was analyzed using qRT-PCR, and it was discovered that alcohol could significantly increase *Gata4* mRNA expression during pregnancy in mice, but anacardic acid could reverse the situation. *: P<0.01 vs. control group (n = 6), **: P<0.01 vs. alcohol group (n = 6).

Quantitative RT-PCR data (**Figure 8**) showed that expression of *Gata4* mRNA in the alcohol group was higher than that in the control group at E14.5 (1.32±0.11 vs. 0.72±0.10, P<0.01). This observation was paralleled by a relative increase in the expression of mRNA and the acetylation of H3K9 to the promoter of *Gata4* on E14.5. Interestingly, anacardic acid could reduce the over-expression of *Gata4* mRNA that was caused by alcohol (anacardic acid + alcohol group vs. alcohol group  = 0.63±0.11 vs. 1.32±0.11, P<0.005). However, the expression of *Gata4* mRNA in DMSO + alcohol group has unaltered significantly compared to the alcohol group (P>0.05).

### Anacardic acid attenuates over-expression of cardiac downstream genes induced by alcohol

**Figure 9:**
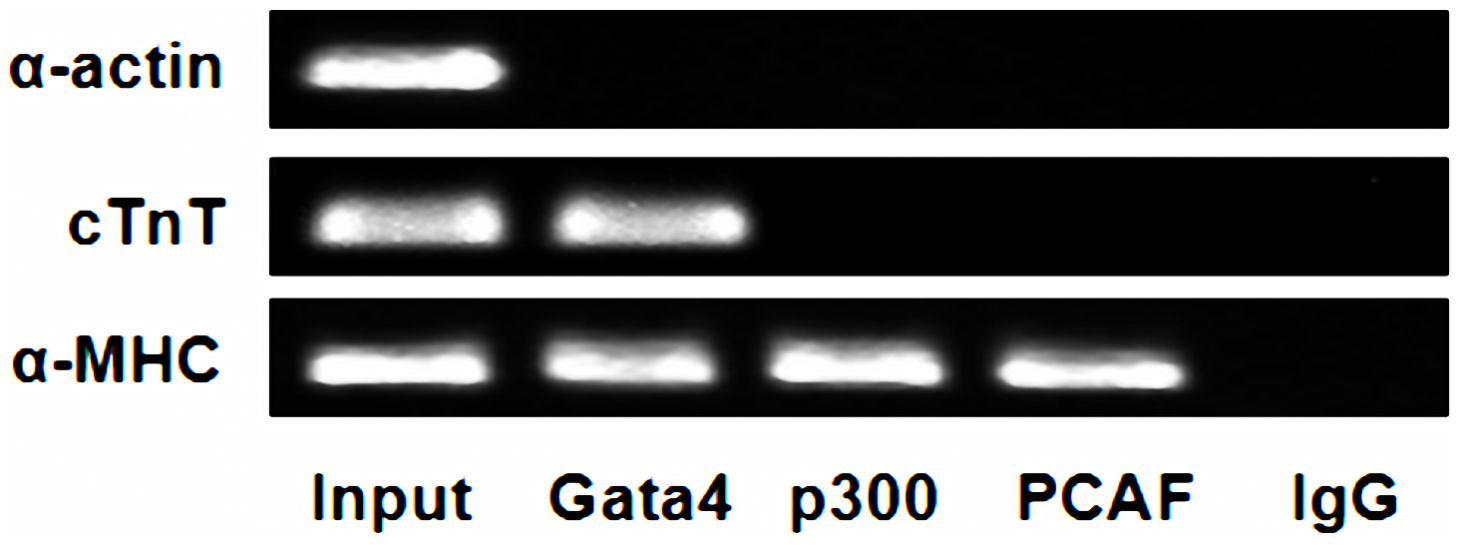
Binding of P300, PCAF and Gata4 protein to the promoter of cardiac downstream genes. ChIP-PCR results showed that the promoter of *α-MHC* allow P300, PCAF and Gata4 proteins to bind, while the promoter of *cTnT* only allows the binding of Gata4. However, P300, PCAF and Gata4 protein could not bind to the promoter of *α-actin*. The Gata4, P300 and PCAF were amplified with DNA fragments precipitated by their respective antibodies (anti-Gata4, anti-P300, anti-PCAF). On the other hand, Input: amplified with DNA fragment without antibody precipitation after sonication and IgG was amplified with DNA fragment precipitated by normal mouse IgG.

**Figure 10:**
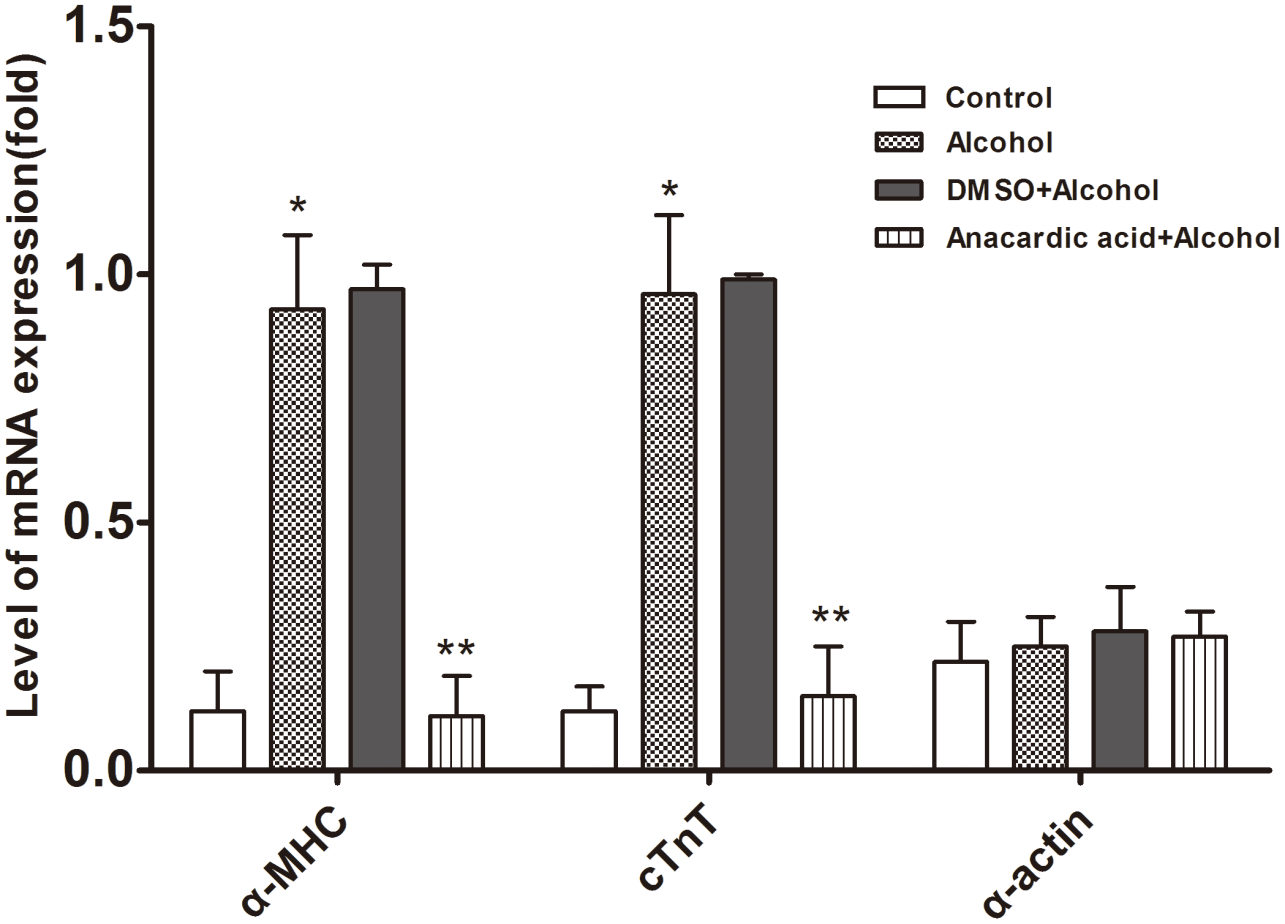
Anacardic acid attenuates over-expression of *α-MHC* and *cTnT* mRNA induced by alcohol. The qRT-PCR study was done to find the mRNA-expression in the mice heart, and a significant increase in *α-MHC* and *cTnT* was noted under alcohol treatment during pregnancy with no effect on *α-actin* mRNA expression. Meanwhile, anacardic acid could reverse mRNA over-expression induced by alcohol of *α-MHC* and *cTnT*. *: P<0.01 vs. control group (n = 6), **: P<0.01 vs. alcohol group (n = 6).

**Figure 11:**
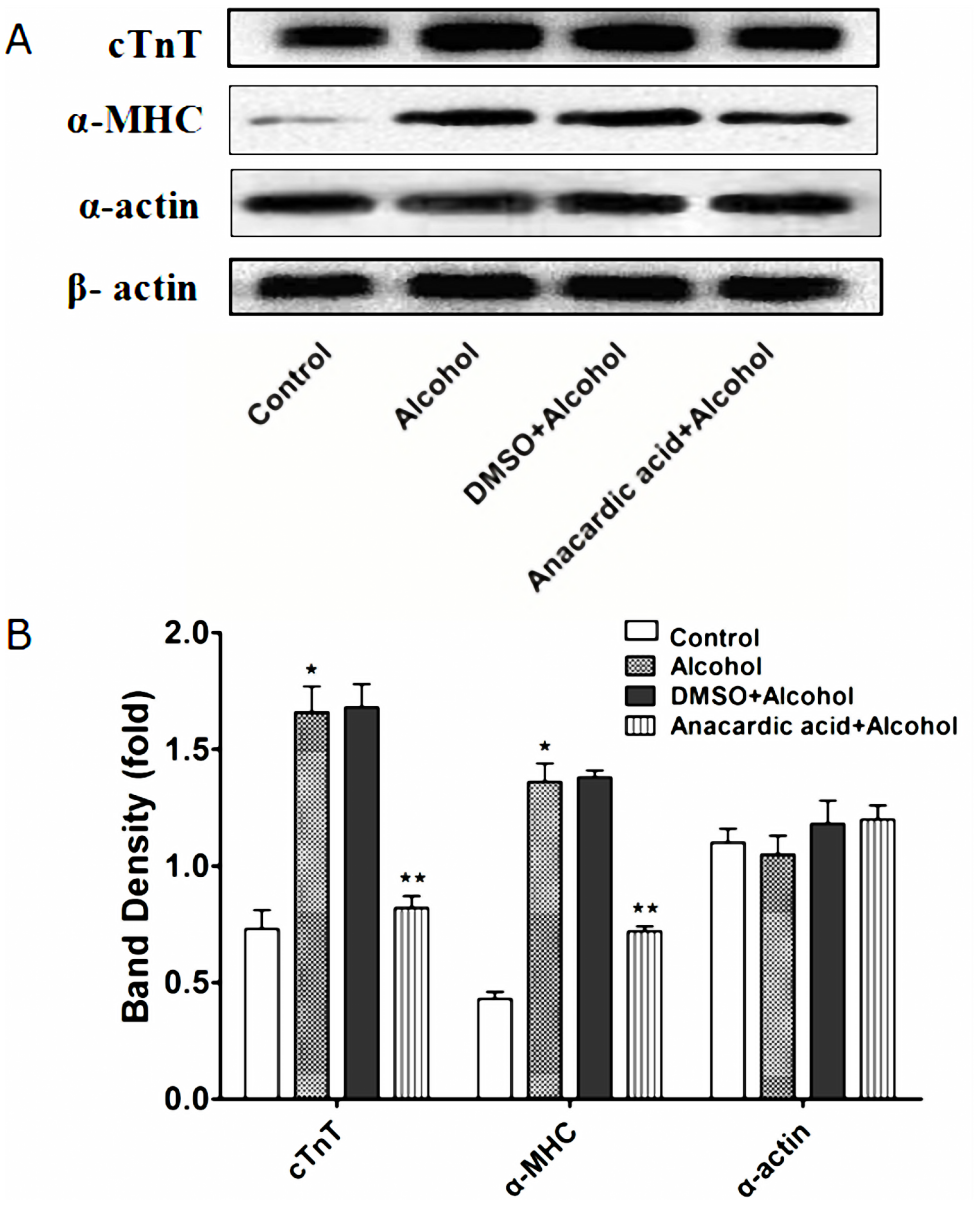
Inhibitory effects of anacardic acid on the over-expression of α-MHC and cTnT protein induced by alcohol. The protein expression of α-MHC, cTnT and α-actin was analyzed using western blot that showed that alcohol could significantly increase α-MHC and cTnT expression at the level of protein in the mice heart exposed to alcohol during pregnancy. Meanwhile, anacardic acid could reverse over-expression induced by alcohol of α-MHC and cTnT protein. However, alcohol could not change the α-actin protein expression in the same samples. *: P<0.05 vs. control group (n = 6), **: P<0.05 vs. alcohol group (n = 6). (A): band density, (B): statistic analysis.

ChIP-PCR data (**Figure 9**) showed that P300 and PCAF could bind to the promoter of cardiac downstream gene *α-MHC*, and Gata4 protein could also bind to the promoter of *α-MHC* and *cTnT*. However, P300, PCAF and Gata4 protein all could not bind to the promoter of cardiac downstream gene *α-actin*. Quantitative RT-PCR data (**Figure 10**) and western blot data (**Figure 11**) showed that alcohol exposure could lead to over-expression of cardiac downstream genes *α-MHC* and *cTnT* at the transcriptional and translational level in the fetal mouse hearts (P<0.01). Note worthily, anacardic acid treatment to alcohol exposed mice could reverse the over-expression of *α-MHC* and *cTnT* (P<0.01). However, the expression of cardiac downstream gene *α-actin* have no obvious change in the fetal mouse hearts exposed to alcohol (P>0.05).

## Discussion

*Gata4* expression is essential for heart development, and any abnormality has been found to cause several cardiac defects. According to some studies, histone acetylases and histone deacetylases are involved in cardiogenesis and myocardial hypertrophy [16]. HATs and HDACs govern gene expression patterns after being recruited to target genes in association with specific transcription factors [17], [18]. However, the roles of HATs and HDACs play to induce over-expression of *Gata4* after alcohol exposure are still unknown. Here we observed that alcohol can increase global HAT activities *in vivo*, but there was no effect on global HDAC activities. To date, five HATs isoforms have been identified in mammalian hearts cells: P300, CBP, PCAF, SRC1 and GCN5 [19]. Some studies have found that steroid receptor co-activators are important transcriptional modulators that regulate nuclear receptor and transcription factors activity. These co-activators are associated with numerous pathologies including cancer, inflammation and metabolic disorders [20]. GCN5 and CBP are the critical transcriptional activators for the regulation and expression of many downstream genes [21]–[24], while PCAF is involved in the pathogenesis of cardiac dysplasia [15].

We have observed that P300, CBP, PCAF, SRC1 and GCN5 all are involved in mediating histone H3K9 acetylation on the promoter of *Gata4* during cardiogenesis. The up-stream domain (∼1,000 bp) of mouse *Gata4* is highly homologous among mouse, rat and human. We also revealed several putative regulatory domains (H3K9 acetylation sites) and binding sites (P300, CBP, PCAF, SRC1, GCN5, etc.) based on our analysis of the sequence. Although there are multiple binding sites of P300, CBP, PCAF, SRC1 and GCN5 on the first 1,000 bp at upstream of *Gata4*, the 43801992 to 43802137 site can specifically be bound by all HATs (P300, CBP, PCAF, SRC1 and GCN5) in cardiac tissues, and histone H3K9 acetylation occur at this site. Therefore, we believe that this site may be a key site of regulating acetylation on the promoter of *Gata4*.

Heterozygous mutation in *Gata4* are known to cause familial septal defects [25]. However, there are many CHD cases that are not related to *Gata4* gene mutations. Spatiotemporal disorder of gene expression pattern caused by genes or environmental factors during early heart development can lead to cardiac dysplasia, but the underlying mechanism(s) remain unclear. Epigenetic mechanisms play a key role in the regulation of embryonic development, tissue homeostasis and modulate cardiovascular diseases [26]. Histones can be modified by several post-translational mechanisms including acetylation, methylation, phosphorylation, ubiquitination, sumoylation and ribosylation of distinct amino acids, and as a consequence either activation or suppression of gene expression [27]–[31]. It is well known that alcohol is a common teratogenic factor during pregnancy and may cause heart malformations during embryonic development, but little is known about the pathogenesis. Histone acetylation represents a central mechanism that control the gene expression [32]. To further explore the specific regulatory roles and the underlying mechanism of HAT (P300, CBP, PCAF, SRC1 and GCN5) mediated cardiac genes regulation, we applied chromatin immunoprecipitation (ChIP) to probe the relationship between HATs and *Gata4* in pregnant mice exposed to alcohol.

*Gata4* is a key transcription factor which is involved in the heart development and cardiac hypertrophy [16]. Many studies have shown that alcohol can enhance the binding of P300, CBP, PCAF and SRC1 on the *Gata4* promoter, and increase histone H3K9 acetylation in association with the *Gata4* promoter. However, the binding of GCN5 on the *Gata4* promoter had no effect on fetal hearts exposed to alcohol. Interestingly, this observation is paralleled by a relative increase in the expression of *Gata4* mRNA and the acetylation of histone H3K9 that binds with the *Gata4* promoter. These results suggested that during alcohol induced over-expression of *Gata4*, P300, CBP, PCAF and SRC1 have effects on *Gata4* expression together with acetylation-dependent modifications to expression of other heart development related genes.

In order to further demonstrate which HAT isoforms (P300, CBP, PCAF, or SRC1) are key *Gata4* regulatory factors, we co-treated alcohol exposed mice with the pan-acetylase inhibitor anacardic acid. Our results demonstrated that anacardic acid can significantly decrease the binding of P300 and PCAF to the *Gata4* promoter. However, binding of SRC1 and CBP was unchanged. Only down-regulation of P300 and PCAF binding reduced hyperacetylation of histone H3K9 induced by alcohol, and this down-regulation played a significant role in the over-expression of *Gata4* induced by alcohol. This suggests that P300 and PCAF may be key regulatory factors that mediate histone H3K9 hyperacetylation and subsequent over-expression of *Gata4* induced by alcohol.

We also found that P300 and PCAF could bind to the cardiac *α-MHC* promoter. In addition, Gata4 protein was co-immunoprecipitate with *α-MHC* and *cTnT* demonstrating that P300, PCAF and Gata4 all take part in regulating the expression of cardiac downstream genes. Our study further demonstrated that alcohol exposure could lead to over-expression of cardiac downstream genes (*α-MHC* and *cTnT*) in the fetal mouse hearts. Importantly, anacardic acid inhibited over-expression of *α-MHC* and *cTnT* induced by alcohol. From this point of view, our study may offer guidance for future advancement of epigenetic medications targeted at preventing and decreasing morbidity and mortality of CHD.

In this regard, reversibility of histone acetylation modification presents a new opportunity for the prevention and treatment of CHD. We observed that the ratios of abortions, stillbirths and intestinal tympanites declined in mice treated with anacardic acid. But this work is preliminary and will be the subject of future studies. Anacardic acid is not approved for human use and little is known regarding its effectiveness and safety in the human body. We believe that is an urgent need for further research into the effectiveness and safety of HATs inhibitor (anacardic acid), with special attention focused on their effects on cardiovascular diseases.

In conclusion, our study indicated that HATs (P300, CBP, PCAF, SRC1 and GCN5) can bind with different sites on the *Gata4* promoter under physiological conditions. Covalent modification imbalance of histone H3K9 acetylation mediated by HATs (P300, PCAF) in the 43801992 to 43802137 region of the *Gata4* promoter may be regarded as a fundamental factor for the control of expression of *Gata4* in the heart of fetal mice exposed to alcohol. These results have highlighted the regulatory mechanism of *Gata4* from the view of epigenetics. Epigenetic regulation may provide novel entry points for therapeutic control of CHD.

## References

[1] van der Linde D, Konings EE, Slager MA, Witsenburg M, Helbing WA, et al (2011) Birth prevalence of congenital heart disease worldwide: a systematic review and meta-analysis. J Am Coll Cardiol 58: 2241–2247. 22078432 10.1016/j.jacc.2011.08.025

[2] Nemer M (2008) Genetic insights into normal and abnormal heart development. Cardiovasc Pathol 17: 48–54. 18160060 10.1016/j.carpath.2007.06.005

[3] Sun YV, Lazarus A, Smith JA, Chuang YH, Zhao W, et al (2013) Gene-specific DNA methylation association with serum levels of C-reactive protein in african americans. PLOS ONE 8: e73480 10.1371/journal.pone.0073480 23977389 PMC3747126

[4] Hon GC, Rajagopal N, Shen Y, McCleary DF, Yue F, et al (2013) Epigenetic memory at embryonic enhancers identified in DNA methylation maps from adult mouse tissues. Nat Genet 45: 1198–1206. 23995138 10.1038/ng.2746 PMC4095776

[5] Connelly JJ, Cherepanova OA, Doss JF, Karaoli T, Lillard TS, et al (2013) Epigenetic regulation of COL15A1 in smooth muscle cell replicative aging and atherosclerosis. Hum Mol Genet 22: 5107–5120. 23912340 10.1093/hmg/ddt365 PMC3842173

[6] Yu CE, Cudaback E, Foraker J, Thomson Z, Leong L, et al (2013) Epigenetic signature and enhancer activity of the human APOE gene. Hum Mol Genet 22: 5036–5047. 23892237 10.1093/hmg/ddt354 PMC3836480

[7] O'Leary CM, Elliott EJ, Nassar N, Bower C (2013) Exploring the potential to use data linkage for investigating the relationship between birth defects and prenatal alcohol exposure. Birth Defects Res A Clin Mol Teratol 97: 497–504. 23873815 10.1002/bdra.23142

[8] Wang E, Sun S, Qiao B, Duan W, Huang G, et al (2013) Identification of functional mutations in GATA4 in patients with congenital heart disease. PLOS ONE 8: e62138 10.1371/journal.pone.0062138 23626780 PMC3633926

[9] Misra C, Sachan N, McNally CR, Koenig SN, Nichols HA, et al (2012) Congenital heart disease-causing Gata4 mutation displays functional deficits in vivo. PLoS Genet 8: e1002690 10.1371/journal.pgen.1002690 22589735 PMC3349729

[10] Zhang Y, Li L, Hua Y, Nunn JM, Dong F, et al (2012) Cardiac-specific knockout of ET(A) receptor mitigates low ambient temperature-induced cardiac hypertrophy and contractile dysfunction. J Mol Cell Biol 4: 97–107. 22442497 10.1093/jmcb/mjs002 PMC3612005

[11] van Berlo JH, Aronow BJ, Molkentin JD (2013) Parsing the roles of the transcription factors GATA-4 and GATA-6 in the adult cardiac hypertrophic response. PLOS ONE 8: e84591 10.1371/journal.pone.0084591 24391969 PMC3877334

[12] Zhong L, Zhu J, Lv T, Chen G, Sun H, et al (2010) Ethanol and its metabolites induce histone lysine 9 acetylation and an alteration of the expression of heart development-related genes in cardiac progenitor cells. Cardiovasc Toxicol 10: 268–274. 20811785 10.1007/s12012-010-9081-z

[13] Wang L, Sun H, Pan B, Zhu J, Huang G, et al (2012) Inhibition of histone acetylation by curcumin reduces alcohol-induced expression of heart development-related transcription factors in cardiac progenitor cells. Biochem Biophys Res Commun 424: 593–596. 22776204 10.1016/j.bbrc.2012.06.158

[14] Yuan JS, Wang D, Stewart CN Jr (2008) Statistical methods for efficiency adjusted real-time PCR quantification. Biotechnol J 3: 112–123. 18074404 10.1002/biot.200700169

[15] Colussi C, Rosati J, Straino S, Spallotta F, Berni R, et al (2011) Nepsilon-lysine acetylation determines dissociation from GAP junctions and lateralization of connexin 43 in normal and dystrophic heart. Proc Natl Acad Sci U S A 108: 2795–2800. 21282606 10.1073/pnas.1013124108 PMC3041095

[16] Yoshida Y, Morimoto T, Takaya T, Kawamura T, Sunagawa Y, et al (2010) Aldosterone signaling associates with p300/GATA4 transcriptional pathway during the hypertrophic response of cardiomyocytes. Circ J 74: 156–162. 19966502 10.1253/circj.cj-09-0050

[17] Kawamura T, Ono K, Morimoto T, Wada H, Hirai M, et al (2005) Acetylation of GATA-4 is involved in the differentiation of embryonic stem cells into cardiac myocytes. J Biol Chem 280: 19682–19688. 15764815 10.1074/jbc.M412428200

[18] Takaya T, Kawamura T, Morimoto T, Ono K, Kita T, et al (2008) Identification of p300-targeted acetylated residues in GATA4 during hypertrophic responses in cardiac myocytes. J Biol Chem 283: 9828–9835. 18252717 10.1074/jbc.M707391200

[19] Yang XJ, Seto E (2007) HATs and HDACs: from structure, function and regulation to novel strategies for therapy and prevention. Oncogene 26: 5310–5318. 17694074 10.1038/sj.onc.1210599

[20] Stashi E, Wang L, Mani SK, York B, O'Malley BW (2013) Research resource: loss of the steroid receptor coactivators confers neurobehavioral consequences. Mol Endocrinol 27: 1776–1787. 23927929 10.1210/me.2013-1192 PMC3787127

[21] Borah JC, Mujtaba S, Karakikes I, Zeng L, Muller M, et al (2011) A small molecule binding to the coactivator CREB-binding protein blocks apoptosis in cardiomyocytes. Chem Biol 18: 531–541. 21513889 10.1016/j.chembiol.2010.12.021 PMC3103858

[22] Chen L, Wei T, Si X, Wang Q, Li Y, et al (2013) Lysine acetyltransferase GCN5 potentiates the growth of non-small cell lung cancer via promotion of E2F1, cyclin D1, and cyclin E1 expression. J Biol Chem 288: 14510–14521. 23543735 10.1074/jbc.M113.458737 PMC3656305

[23] Tian X, Zhao F, Cheng Z, Zhou M, Zhi X, et al (2013) GCN5 acetyltransferase inhibits PGC1alpha-induced hepatitis B virus biosynthesis. Virol Sin 28: 216–222. 23913178 10.1007/s12250-013-3344-3 PMC8208399

[24] Downey M, Knight B, Vashisht AA, Seller CA, Wohlschlegel JA, et al (2013) Gcn5 and sirtuins regulate acetylation of the ribosomal protein transcription factor Ifh1. Curr Biol 23: 1638–1648. 23973296 10.1016/j.cub.2013.06.050 PMC3982851

[25] Rajagopal SK, Ma Q, Obler D, Shen J, Manichaikul A, et al (2007) Spectrum of heart disease associated with murine and human GATA4 mutation. J Mol Cell Cardiol 43: 677–685. 17643447 10.1016/j.yjmcc.2007.06.004 PMC2573470

[26] Ohtani K, Dimmeler S (2011) Epigenetic regulation of cardiovascular differentiation. Cardiovasc Res 90: 404–412. 21372004 10.1093/cvr/cvr019

[27] Klose RJ, Zhang Y (2007) Regulation of histone methylation by demethylimination and demethylation. Nat Rev Mol Cell Biol 8: 307–318. 17342184 10.1038/nrm2143

[28] Kouzarides T (2007) Chromatin modifications and their function. Cell 128: 693–705. 17320507 10.1016/j.cell.2007.02.005

[29] Li B, Carey M, Workman JL (2007) The role of chromatin during transcription. Cell 128: 707–719. 17320508 10.1016/j.cell.2007.01.015

[30] Cedar H, Bergman Y (2009) Linking DNA methylation and histone modification: patterns and paradigms. Nat Rev Genet 10: 295–304. 19308066 10.1038/nrg2540

[31] Rando OJ, Chang HY (2009) Genome-wide views of chromatin structure. Annu Rev Biochem 78: 245–271. 19317649 10.1146/annurev.biochem.78.071107.134639 PMC2811691

[32] Peng Y, Lambert AA, Papst P, Pitts KR (2009) Agonist-induced nuclear export of GFP-HDAC5 in isolated adult rat ventricular myocytes. J Pharmacol Toxicol Methods 59: 135–140. 19328241 10.1016/j.vascn.2009.03.002

